# Use of general practitioner services among youth and young adults in Norway from 2006 to 2021

**DOI:** 10.1080/02813432.2023.2280045

**Published:** 2023-11-29

**Authors:** Kirsti Wahlberg, Kristine Pape, Bjarne Austad, Gunnhild Åberge Vie

**Affiliations:** a Department of Public Health and Nursing, Faculty of Medicine and Health Sciences, Norwegian University of Science and Technology, NTNU, Trondheim, Norway; b General practice research unit, Department of Public Health and Nursing, Faculty of Medicine and Health Sciences, Norwegian University of Science and Technology, NTNU, Trondheim, Norway

**Keywords:** adolescent, young adult, health services research, primary health care, general practice

## Abstract

**Objective:**

To describe the frequency and content of contacts with general practitioners (GPs) among youth and young adults by sex, age and time, emphasizing mental health, sexual health and respiratory tract infections.

**Design:**

Registry-based population-wide cohort study.

**Setting:**

General practice in Norway 2006–2021.

**Subjects:**

Norwegian residents aged 13–25 within the study period.

**Main outcome measures:**

Contacts with GPs and out-of-hours services, including type of contact, specific procedures and diagnoses.

**Results:**

Average number of GP consultations increased over the study period for all age groups. Conversation therapy and time-consuming consultations increased over time, while chlamydia testing and contraceptive guidance decreased among young women. Consultations with mental health diagnoses increased substantially over the study period for all age groups. Use of GP and out-of-hours services increased with age, with a peak at the end of upper secondary school. Youth more often met their own regular GP when consulting for mental health diagnoses than for respiratory tract infections.

**Conclusion:**

This study confirmed the continuing trend of increasing use of general practice services among youth, with an increase in conversation therapy and consultations with mental health diagnoses. Procedures related to sexual health became less common. Youth usually meet their regular GP for consultations, in particular those whose diagnosis indicates the highest need of continuity.

## KEY POINTS

- Youth are a healthy group with relatively low GP use. However, consultation rates have been increasing in later years.
- We found that consultation rates increased more over time than can likely be attributed to known policy changes.
- Consultations with mental diagnoses and conversational therapy increased substantially among youth aged 13–25, while family planning consultations and related procedures decreased.
- The youth usually meet their own regular GP, particularly if they have diagnoses indicating higher need for follow-up.

## Background

Adolescents and young adults in Norway have low levels of healthcare use and low mortality [1,2]. However, the prevalence of anxiety and depressive disorders, overweight and obesity as well as the use of analgesics has increased among young people [3–5]. Poor mental health is particularly concerning, as it is associated with poorer economic, social and health outcomes in early adulthood [6].

The Norwegian regular general practitioner (GP) scheme ensures that everyone is entitled to a specific primary care physician (hereafter referred to as ‘regular GP’) [7]. The regular GP is responsible for providing all primary care physician services to their list of patients (i.e. patients who have chosen the specific GP as their regular GP) and acts as a gatekeeper to the specialist health care, referring the patient when needed. The regular GPs offer continuity of health care throughout the life course, including preventive medicine and coordination of health care [8]. Personal continuity of care may play an important role in aiding the transition from child to adult health care [9]. In addition to the publicly funded GP scheme, municipalities offer free and easily available preventive health care for children and adolescents, mainly staffed by public health nurses [10]. GPs are a cornerstone in the Norwegian health care system, offering treatment at the lowest effective level [7].

Young people consult a GP less often than adults but have had a trend of increasing GP consultations from 2010 until the COVID-19 pandemic [2]. The reasons for such trends may include changes within individual factors (such as level of illness), organization and availability of health services, societal norms and technological innovations [11]. In 2016, an absence policy was introduced in upper secondary schools, requiring a medical certificate in case of school absence exceeding 10%. It resulted in increased consultations for ages 16–19 [12], and there was a decrease during the COVID-19 pandemic, when the policy was temporarily reversed [2]. Also removal of copayments has been shown to contribute to increased GP use for adolescents, as was the case in Norway in 2010 when the age limit for copayments was moved from 12 to 16 years [13, 14]. Other factors shown to stimulate the consultation rate in adolescents and young adults are the GP’s free capacity [15] and campaigns to promote GP use [16]. However, there have been no such campaigns during the last decade, and the free capacity of GPs has decreased.

Youth and adolescents’ use of GP services has previously been studied for specific diagnoses or population subgroups, and mental health and respiratory tract infections are found to be common [17–19]. Data on GP contacts over time are publicly available but only for broader age groups [2,20]. With time, the level of personal continuity as well as the GP’s role in youth’s primary health care use might change, and procedures performed by GPs have rarely been included in previous Norwegian studies. Also, few papers have focused on GP continuity for the youth population, one study found 69% of consultations to be with regular GP for 15–24-year-olds [21]. A comprehensive overview of trends in youths’ contacts with GPs is thus lacking.

We aimed to describe the use of GP services in youth and young adults over a period of 15 years. We describe contacts, diagnoses and procedures by age, sex and year.

## Methods

### Study design

We performed a longitudinal register study of Norwegians aged 13–25. The data consisted of all reimbursement claims from Norwegian GP and out-of-hours (OOH) offices from 2006 to 2021.

### Setting

Each regular GP’s list has a limit for the maximum number of patients assigned to it. Most GPs work in group practices (any GP practice hereafter referred to as “GP practices”), including both regular GPs (list-owners) and sometimes their locums and interns. Emergency contacts within office hours are usually handled at the GP practice, while the OOH services provide emergency primary care outside office hours [7].

In several GP practices, GP’s share the acute appointments between them on separate days, thus we assumed the probability of meeting your regular GP would be lower for more acute diagnoses.

Funding of GP services organised within the public healthcare sector is partly based on activity-based reimbursement claims. These claims include codes for the type of contact and procedures performed [22] and diagnosis codes are classified by the international classification for primary care (ICPC-2) [23].

Norwegian children and youth are entitled to primary and secondary education. Secondary school is divided into lower (years 8–10) and upper secondary school (years 11-13). Lower secondary school is mandatory and lasts from age 13 to 16. Upper secondary school is divided into general studies lasting 3 years, and vocational studies typically lasting 2 years with an additional 2 years of apprenticeship [24].

### Study population

We included all individuals on January 1^st^ the year they turned 13 and excluded them from January 1^st^ the year they turned 26. Individuals were included or re-included the day they immigrated and excluded at day of emigration or death.

### Data sources

Statistics Norway provided population statistics, including date of death and immigration/emigration. Information on publicly funded primary health care services use (GP ID, patient ID, time, diagnoses, contact type and procedures from reimbursement claims) was collected from the Control and Payment of Health Reimbursement Register (KUHR). Information about registered regular GP was collected from the Norwegian GP Registry. These three registries were linked by ID and time.

The project has been approved by the regional ethics committee (REK Midt) number 2016/2159 and later expansions. Informed consent was not deemed necessary by REK.

### Variables

#### Outcomes

We identified three main outcomes: 1) type of contact and context (GP practices or OOH services), 2) procedures performed during consultations at the GP practices and 3) diagnoses from consultation reimbursement claims from the GP practices.

We counted a number of contacts per half year (and subsequently merged to per year for some analyses) for the following contact types: consultations, simple contacts, home visits and interdisciplinary contacts, see Table 1.

**Table 1.** Contact types based on the grouping by the Norwegian Directorate of health [20] and the seven selected procedures. *code implemented in 2010 **code implemented in 2014.

Outcome	Meaning	Reimbursement codes
Contact types		
Consultation	Doctor’s appointment where the patient met the doctor either physically or for an electronic appointment	2ad, 2ae, 2ak, 2aek, 2ed, 2fk, 2af
Simple contact	Shorter contact with the GP practice or OOH services; physically, electronically or by phone	1ad, 1ak, 1bd, 1be, 1bk, 1e, 1 g, 1h, 1i
Home visit	Consultations outside of GP practice or OOH services (at the patients home)	11ad, 11ak
Interdisciplinary	Contacts between GP and other care personnel, with or without the patient’s attendance	1f, 14, 1j
Procedures		
Chlamydia tests	A chlamydia test was performed	705h
Contraceptive guidance	Guidance on possible contraceptive methods for the patient, not necessarily continued prescription	110*
Gynaecological exam	A gynaecological examination has been performed during the consultation	103b**
Conversational therapy	A therapeutic conversation with a patient with mental disorders	615
Conversation with next of kin	A necessary conversation with next of kin about certain problems or diagnoses	612a, 612b
Time-consuming referral	A fee for time spent for writing certain referrals	616
Consultation >20 min	Consultation lasting more than 20 min	2cd, 2ck

We chose respiratory tract infections, mental health and sexual health as three important health topics, relevant for young people visiting a GP practice. Respiratory tract infections and mental diagnoses are frequent amongst this age group and both mental and sexual health can have large impacts on youths’ everyday life.

We identified seven specific procedures (based on their reimbursement codes) selected because of their relevance for sexual or mental health, see Table 1 [22]. We dichotomized these procedures to whether they had been performed or not per year.

We defined diagnosis groups based on grouping by Statistics Norway [25], also dichotomized to yes/no per year. 1) Respiratory tract infections (including otitis and COVID-19 diagnoses), 2) Mental health diagnoses, 3) Family planning (pregnancy/birth/contraception) and 4) Female genitalia.

For every GP consultation at GP practices, we also identified whether the treating GP (the one making the claim) was the same as the registered regular GP of that patient for that month.

#### Covariates and subgroups

We calculated age as year of contact minus year of birth, as the exact date of birth was not available in our data set. The first half of the year they turned 13 we named 13.0, the second half of the same year 13.5 etc., differentiating between spring semesters and autumn semesters. For analyses of time trends, we categorized age as 13–15, 16–19 and 20–25 corresponding approximately to lower secondary school, upper secondary school and after secondary school, respectively. Immigration status was categorized by Statistics Norway according to the country of birth for the youth and his/her parents [25].

For the assessment of whether the patient met the regular GP, we defined subgroups of patients with rare or frequent GP visits (<0.5 versus >5 visits/year) and defined three diagnosis groups of interest, respiratory tract infections, mental health diagnoses and lastly family planning and female genitalia diagnoses combined.

Respiratory tract infections reflect an acute need for health care, while mental diagnoses are diagnoses for which patients would benefit from personal continuity from their regular doctor, and possibly needing more follow-up. Family planning and female genitalia were combined as an area where we expect consultations to be less acute than respiratory tract infections; however, with a smaller need for continuity than mental health diagnoses.

### Statistical analyses

We used Stata version 16.0 for all analyses.

We adjusted for immigration status in all analyses by adding it as a categorical variable. Year was included in all statistical models, either as a main exposure, a covariate for adjustment or by restricting analyses to a single year.

We analysed the number of consultations, simple contacts and interdisciplinary contacts to GP practices and the number of consultations and simple contacts to OOH services in 2019 by age using Poisson regression, with standard errors corrected for intrapersonal correlation and an interaction term between age in half years and sex. We chose 2019 as the most recent year unaffected by the COVID-19 pandemic. Similarly, we analysed consultations and simple contacts to GP practices per year using Poisson regression with an interaction term between three age groups and each year. We used the postestimation command margins to estimate adjusted average number of consultations, simple contacts and interdisciplinary contacts and displayed the results graphically. Because the results were estimated per person, the numbers are valid regardless of size of the age group.

For the procedures, we performed clustered GEE analyses with robust standard errors. We used an interaction term between year (categorical) and three age groups, separately for males and females. Similarly, we performed analyses with an interaction term between age (categorical variable) and sex, adjusting for year. Gynaecological examinations and contraceptive guidance were only analysed among females.

We also performed clustered GEE analyses for the chosen diagnosis groups, with robust standard errors. Analyses were performed separately for males and females, and family planning and female genitalia were performed only for females.

We tabulated the probability of seeing the regular GP overall and according to year, consultation frequency and diagnosis group. We additionally performed GEE analyses to estimate the proportion of youth who had any consultation (at GP practices) per half year.

## Results

### Study sample

The data set comprised of 1,876,364 individuals and 13,306,237 observed person years. Individuals were followed for an average of 7.1 years. Data on sex and year of birth were complete. See Table 2 for additional population statistics.

**Table 2.** Population characteristics.

Category	Variable	Proportion (%)
Sex	Male	51.0
Immigration status	Norwegian-born to Norwegian parents	72
	Immigrant	15.0
	Other immigration status	13.0
	Missing	<0.1

### Consultations, simple contacts and interdisciplinary contacts by age

We found few home visits (0.6% of study participants per year), and we therefore chose to further exclude them from the results of this paper.

**Figure 1.**
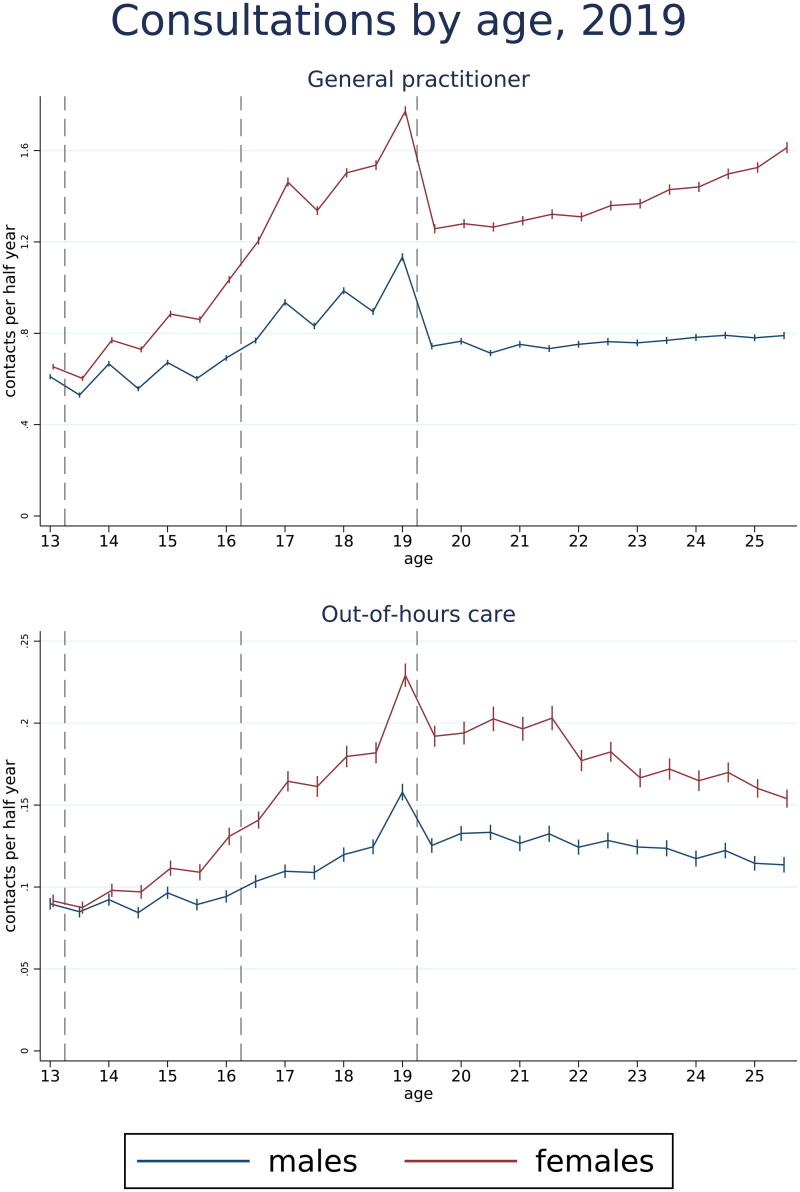
Estimated number of consultations with GP practices and out-of-hours services in 2019 per half year from age 13 to 25 for Norwegian males and females with 95% confidence intervals. Results based on Poisson regression analyses, adjusted for immigration status. Stapled lines indicate transitions between primary, lower and upper secondary school, as well as when students finish upper secondary school if completing a 3-year course as scheduled.

The estimated number of consultations at GP practices per half year for 2019 increased from the age of 13 to 25 for both males and females, from 0.61 (95% CI 0.60–0.62) to 0.79 (95% CI 0.77–0.81) for males and a marked increase from 0.65 (95% CI 0.64–0.67) to 1.61 (95% CI 1.59–1.64) for females, shown in Figure 1. Around the end of upper secondary school, there was a substantial drop in the number of consultations, from 1.13 (95% CI 1.12–1.15) to 0.74 (95% CI 0.73–0.76) per half year for males and from 1.77 (95% CI 1.75–1.79) to 1.26 (95% CI 1.24–1.28) per half year for females. Although less frequent, simple contacts showed a similar trend, with a drop after upper secondary school (supplementary figure S1). Interdisciplinary contacts were substantially less frequent but also increased by age (supplementary figure S4).

The estimated number of OOH consultations increased up until the end of upper secondary school (from 0.09 (95% CI 0.09–0.09) at age 13.0 to 0.16 (95% CI 0.15–0.16) at age 19.0 for males per half year and from 0.09 (95% CI 0.09–0.10) at age 13.0 to 0.23 (95% CI 0.22–0.24) at age 19.0 for females per half year), most markedly for females, after which they decreased slightly (to 0.11 (95% CI 0.11–0.12) per half year for males and 0.15 (95% CI 0.15–0.16) for females), Figure 1. Simple contacts with OOH services also increased until end of upper secondary school, after which they remained stable (supplementary figure S1).

### Consultations, simple contacts and interdisciplinary contacts in GP practices by time

**Figure 2.**
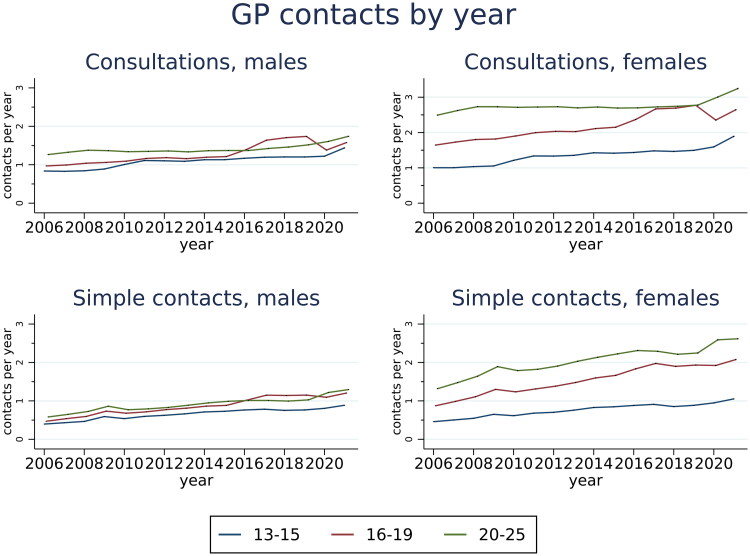
Estimated number of consultations and simple contacts with GP practices among Norwegian youth by sex, age groups and year with 95% confidence intervals. Results are based on Poisson regression analyses, adjusted for immigration status.

Both consultations and simple contacts with GP practices increased from 2006 to 2021 for both sexes and all three age groups (Figure 2). Consultations increased markedly from 2016 in 16–19-year-olds followed by a decrease in 2020, and a similar tendency was seen for simple contacts. Consultations among 13–15-year-olds increased between 2009 and 2011, as well as from 2020 to 2021. Interdisciplinary contacts increased over time, but more markedly from 2019 (supplementary figure S4). The probability of a consultation also increased over time for all age groups and both sexes (supplementary figure S2).

### Procedures at the GP practice

**Figure 3.**
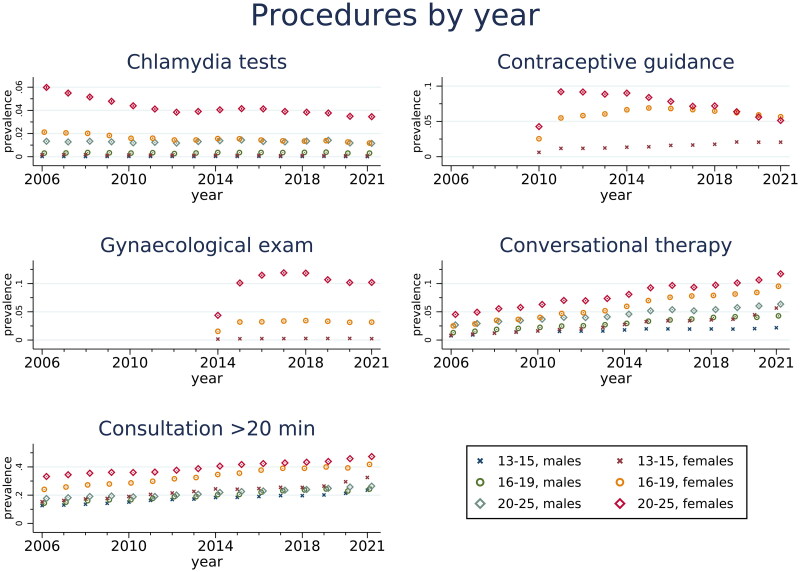
Estimated prevalence of procedures performed at GP practices from 2006 to 2021 for Norwegian males and females aged 13-25 with 95% confidence intervals. Results based on generalized estimating equations with years clustered within individuals. The code for contraceptive guidance was introduced in 2010, for gynaecological examination in 2014.

Figure 3 presents selected procedures performed in the GP’s office from 2006 to 2021, showing the estimated likelihood of a patient of the given sex and age group to have had the stated procedure per year.

We found a decline in chlamydia testing among females 20–25 years old (from 6.0% (95% CI 5.9–6.1) in 2006 to 3.5% (95% CI 3.4–3.5) in 2021) and to a smaller extent also among 16–19-year-olds. Contraceptive guidance decreased for females aged 20–25 (from 9.2% (95% CI 9.1–9.3) in 2011 to 5.1 (95% CI 5.0–5.2) in 2021), while we observed a small increase among 13–15-year-old females. Chlamydia testing among males was low and stable over time.

Conversational therapy and consultations lasting longer than 20 min increased for all age groups. Conversational therapy for 13–15-year-olds increased from 0.8% (95% CI 0.7–0.8) to 2.2% (95% CI 2.1–2.3) for males and from 0.8% (95% CI 0.8–0.9) to 5.7% (95% CI 5.5–5.8) for females between 2006 and 2021. Corresponding numbers for 20–25-year-olds are 2.7% (95% CI 2.6–2.8) to 6.4% (95% CI 6.3–6.5%) for males and 4.5% (95% CI 4.4–4.6%) to 11.7% (95% CI 11.6–11.9) for females.

Time-consuming referrals increased for all age groups by time, while conversations with next of kin increased for the two youngest age groups; however, with a temporary decrease between 2014 and 2018 (supplementary figure S3).

### Diagnoses

**Figure 4.**
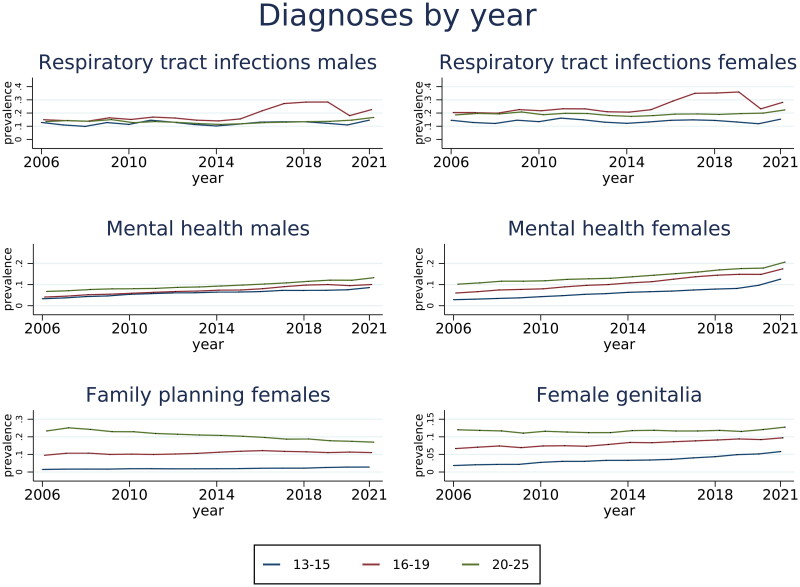
Estimated prevalence of respiratory tract infections, mental health diagnoses, family planning and female genitalia diagnoses, by sex, age groups and year with 95% confidence intervals. Results are based on generalized estimating equations with years clustered within individuals.

The estimated prevalence of respiratory tract infections, mental health diagnoses, family planning and female genitalia diagnoses are presented in Figure 4 by sex, age groups and year. There was an increase in the prevalence of mental health diagnoses with time for both sexes and all age groups. Respiratory tract infections increased substantially from 2016 in the age group 16–19 but remained stable for other age groups. We also note a substantial decrease in diagnoses related to family planning amongst 20–25-year-old females, from 23.3% to 17.0%. Diagnoses of female genitalia increased for 13–19-year olds, from 1.8% to 5.8%.

### Do youth meet their regular GP?

When adolescents had a consultation at the GP’s office, they met their regular GP 63.6% of the times. In 2006, 62.5% of consultations were with the regular GP, 64.2% in 2019, and 69.1% in 2021. For patients who visited on average <0.5 times per year, 59.8% of consultations were with the regular GP, compared to 66.1% of the consultations among patients visiting >5 times/year.

A smaller proportion of consultations were with their regular GP for respiratory tract infections, 55.8%, compared to 75.5% when psychological/psychiatric diagnoses were set. Consultations with diagnoses of family planning and/or female genitalia were with the regular GP in 67.1%.

## Discussion

### Key results

The use of GP services increased since 2006 for all sex and age category subgroups. Consultations increased with age, with a peak at the end of upper secondary school. OOH consultations and simple contacts also increased with age until the end of upper secondary school but decreased and flattened thereafter, respectively. Consultations and procedures related to reproductive health decreased over the study period among young women, except for female genitalia diagnoses. Consultations with mental diagnoses and related procedures increased for all subgroups, as did time-consuming consultations. Young people consulting the GP practices usually met their registered regular GP, in particular those with mental diagnoses and the presumably highest need for continuity.

### Interpretation and comparison with existing literature

The observed increase in GP consultations during upper secondary school corresponds well to the absence policy from 2016, which may force a higher consultation frequency during these school years [12]. In addition, the Norwegian tradition of school leaving celebrations in the last spring of upper secondary school may contribute to the peak in both GP practice and OOH services consultations at age 19 [26]. A possible mechanism for the sudden drop in GP consultations and simple contacts after upper secondary school might be youth moving away from home and, unless they request a change, away from their regular GP. Economic and practical reasons (and living away from family) might give a delay in contact and a slightly increased chance of using OOH services rather than GP practices.

Our finding of increasing consultation frequency over time is in accordance with official statistics and increasing trends are seen in other age groups [2]. In contrast, the consultation rate (of youth aged 10–29) during working hours in Denmark has had a decreasing trend from 2006 until 2019. The 2019 consultation rates were lower among young people in Denmark compared to Norway, although representing somewhat different age groups [27]. This difference is somewhat surprising, as Denmark is a neighbouring country to Norway, with a similar culture and health care system. Our findings cannot be completely attributed to the absence policy or the changes to copayments introduced during this period [12–14], as the increase can be seen over all age groups and not only the groups affected by policy changes. In addition, the Danish proportion of young people attending consultations remained relatively stable [27]. The increasing proportion of young people visiting the GP implies that the increase in consultation rates cannot be driven by more frequent attendance by youth already visiting their GP. Electronic consultations were introduced in 2013, and their use increased substantially during the pandemic [28]. In light of how societal norms and technological innovations can influence healthcare use [11], availability of electronic consultations might have contributed to the increased consultation rate from 2020. However, this is hard to disentangle from the consequences of the pandemic and beyond the scope of this paper.

Mental disorders require more follow-up consultations compared to many other diagnoses experienced by this age group. The observed increase in conversational therapy and mental diagnoses, thus likely contributed to the general increase in GP consultations. Our findings indicate that the GP is important in treatment of mental disorders among the youth and succeeds to provide a certain degree of continuity in the follow-up of such diagnoses. We note a large increase in conversational therapy that indicate that GPs use psychotherapeutic approaches. A key question is whether this increase corresponds to an actual worsening in mental health among youth and thereby an increased need. Recent findings of increased self-reported mental health symptoms from surveys [4], increased prescriptions of antidepressants and anxiolytics [29] and a trend of increasing emotional problems in other countries [30] may support this. However, increasing help-seeking behaviour not corresponding to an actual increased need might lead to overdiagnosis and overtreatment.

Our results show increasing sex differences in attending primary care from age 13 onwards. Danish statistics reveal a similar trend [27]. Several sex-specific health issues appear with the onset of puberty, though they cannot account for all the differences in healthcare use [31].

We found decreasing chlamydia testing and contraceptive guidance, and a decreasing trend of family planning diagnoses although prescription rates for contraceptives have not changed considerably for females aged 15–24 over this period [29]. Chlamydia testing among males has remained low over time. Changes to chlamydia testing procedures in GP practices might also have affected coding practices. Since 2002, Norwegian public health nurses and midwives have obtained more rights to prescribe and administer contraceptives [32], which may have shifted some of the youth’s sexual health consultations away from the GP’s office. Fewer births by mothers aged <25 may also have contributed to fewer family planning consultations [1]. However, diagnoses related to female genitalia have increased for the youngest age groups. This indicates that the doctor still has an important role in treating symptoms and diseases of the female genital tract.

Our findings of respiratory tract infections as a common diagnosis correspond well to both Norwegian and international findings [17, 33]. The increase between 2016 to 2019 with the following decrease for 16–19-year-olds corresponds well to the aforementioned absence limit. However, youth’s attention to, interpretation of and contact seeking for respiratory tract symptoms could have been changed by the pandemic.

In addition to an increase over time in direct contact between GPs and young patients, the present findings also show a similar increase in communication and collaboration related to the patient, both with next of kin (conversations with next of kin) and other professionals (interdisciplinary contacts and time-consuming referrals). This, along with an increased number of contacts, conversational therapy and time-consuming consultations, indicate an increased workload with young patients. This could indicate an increasing discrepancy between demand and availability.

On the other hand, we note that youth usually meet their regular GP, corresponding to earlier findings [21], and with no declining trend. Furthermore, personal continuity seems greater when they attend frequently or have diagnoses that often require follow-up. This suggests that in the majority of cases, GPs are so far able to offer personal continuity to their young patients despite their high workload.

### Strengths and limitations

Our data are almost complete and without recall bias. Our findings also have a certain degree of external validity for similar healthcare systems and populations, as trends of increasing mental health problems are observed internationally [30].

The advantage of using reimbursement claim codes is that the fee received gives physicians an incentive for complete coding of contacts. However, available reimbursement codes (and, to some extent diagnoses) change over time, which might affect the usage of the different codes. For instance, in 2019, tariff 1j was added for consulting a specialist care doctor. We were unable to differentiate between changed coding or changed activity as the reason for the sharp increase in interdisciplinary contacts.

The economic incentive for complete registration does not apply to diagnoses, which might lower their reliability. A study from Norwegian primary care still found a good coherence between ICPC-2-diagnoses and consultation notes [34]. Grouping codes and diagnoses might increase their reliability. Data from municipal school health services, youth health centres, military doctors or fully privately funded physicians was not available. This would have added valuable information on the health service use in this age group.

## Conclusion

This study highlights the importance of the GP for adolescents, a group currently experiencing societal stressors and worsening mental health. The increased service use and especially regarding mental health should be of notice for policymakers and healthcare personnel.

## Supplementary Material

Supplemental Material
