# Supplementary material for: Use of general practitioner services among youth and young adults in Norway from 2006 to 2021
Source: Scand J Prim Health Care. 2023 Nov 29;41(4):505–15. doi: 10.1080/02813432.2023.2280045 (PMC11001332; doi:10.1080/02813432.2023.2280045)
Supplement: Supplemental Material [file IPRI_A_2280045_SM2494.docx]

Use of general practitioner services among youth and young adults in Norway from 2006 to 2021

# Supplementary material

Table S1: Diagnosis groups and included ICPC-2 codes, coded the same way as diagnostic groups defined by Statistics Norway [1].

| Diagnosis group | ICPC-2 codes |
| --- | --- |
| Respiratory tract infections including otitis and covid diagnoses | R05, R09-R23, R71-R83, H71-H74, R991, R992 |
| Mental health diagnoses | P01-P26, P28, P29, P70-P99 |
| Female genitalia | X01-X22, X28, X29, X70-X74, X78-X81, X84-X99 |
| Family planning (pregnancy/birth/contraception) | W01-W03, W05, W10-W15, W17-W19, W21, W27-W29, W70, W71, W73, W75, W76, W78-W85, W90-W96, W99 |

Fig. S1: Estimated number of simple contacts with GP practices and out-of-hours services in 2019 per half year from age 13 to 25 for Norwegian males and females with 95% confidence intervals. Stapled lines indicate transitions between primary, lower and upper secondary school, as well as when students finish upper secondary school if completing a three-year course as scheduled.


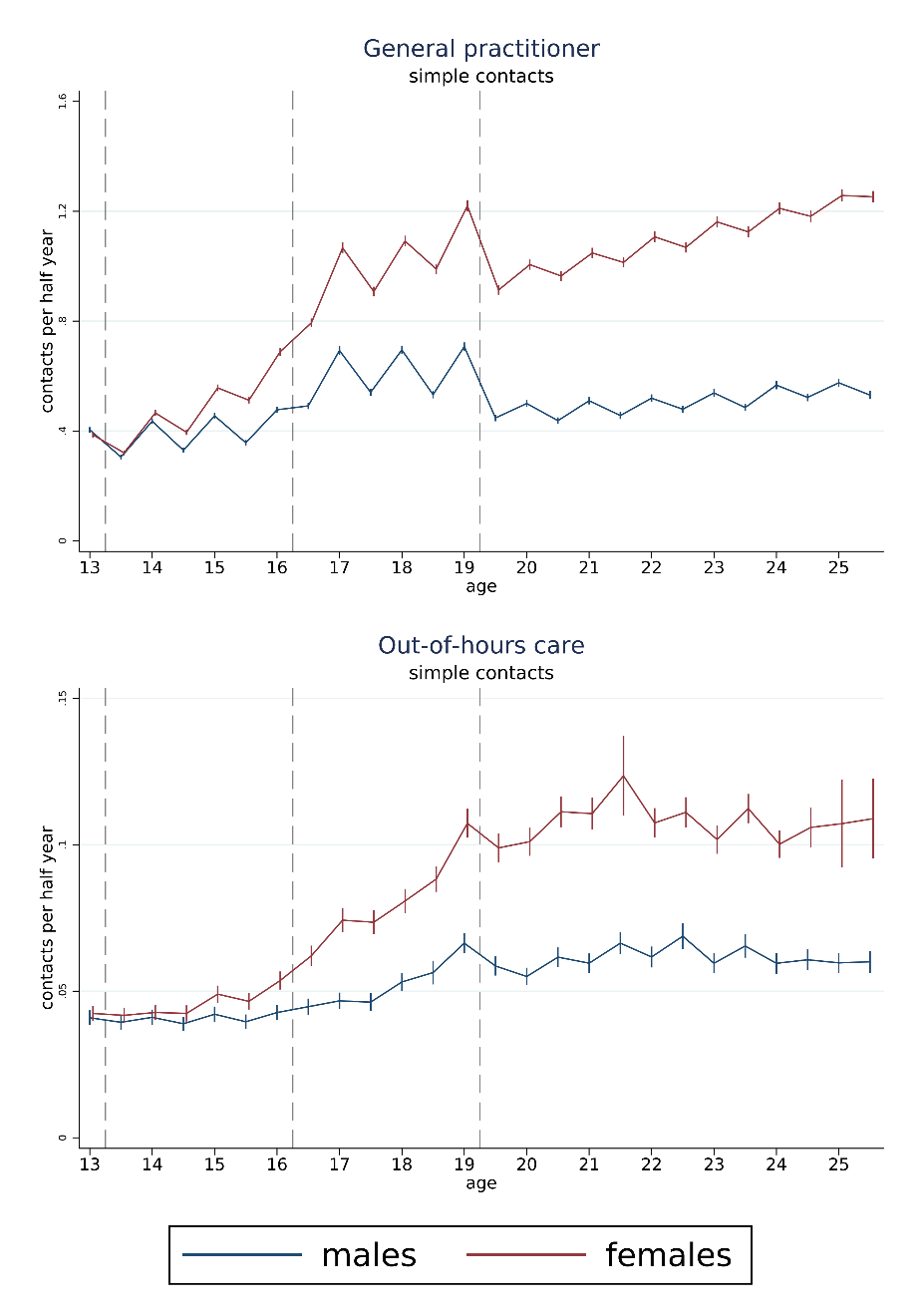


Fig. S2: Probability of consultation with a GP practice per half year for males and females by year and age groups with 95% confidence intervals.


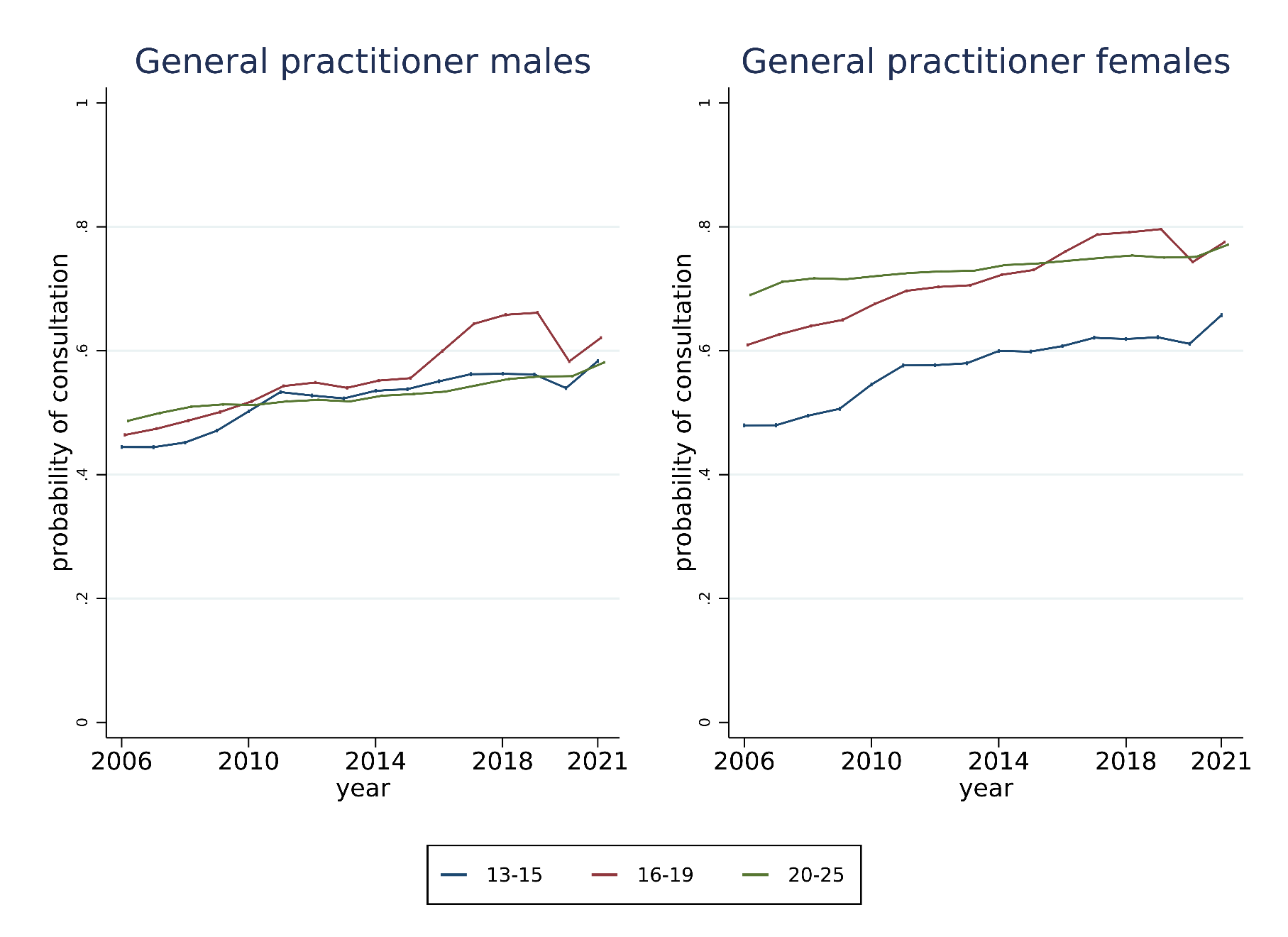


Fig. S3: Use of procedure reimbursement codes for males and females by age groups and year with 95% confidence intervals. Addition to fig. 3


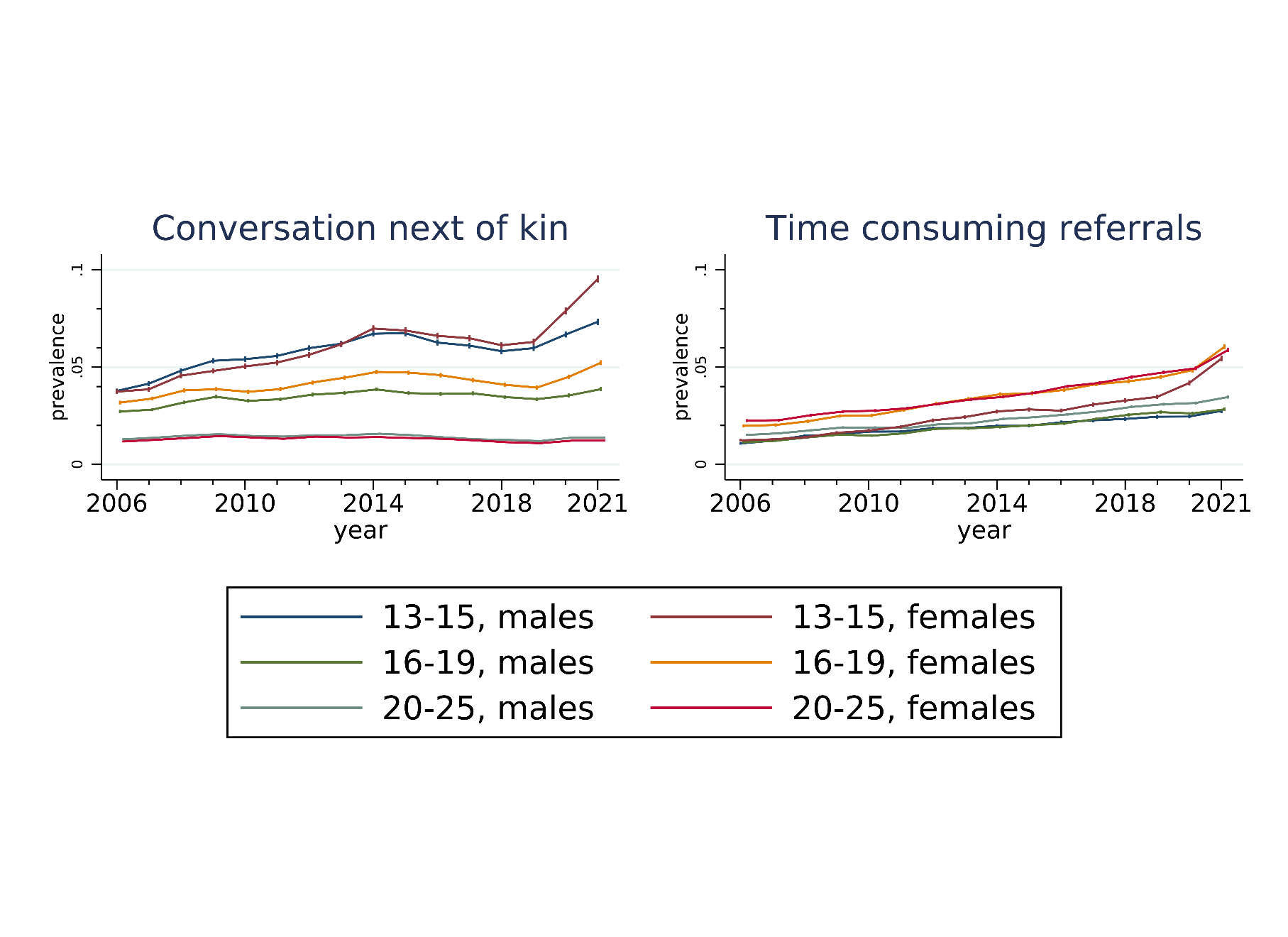


Fig. S4: Estimated number of interdisciplinary contacts with GP practices per half year with 95% confidence intervals. Uppermost and middle estimated for age groups by year. Lowest estimated for 2019 by age. Stapled lines indicate transitions between primary, lower and upper secondary school, as well as when students finish upper secondary school if completing a three-year course as scheduled.


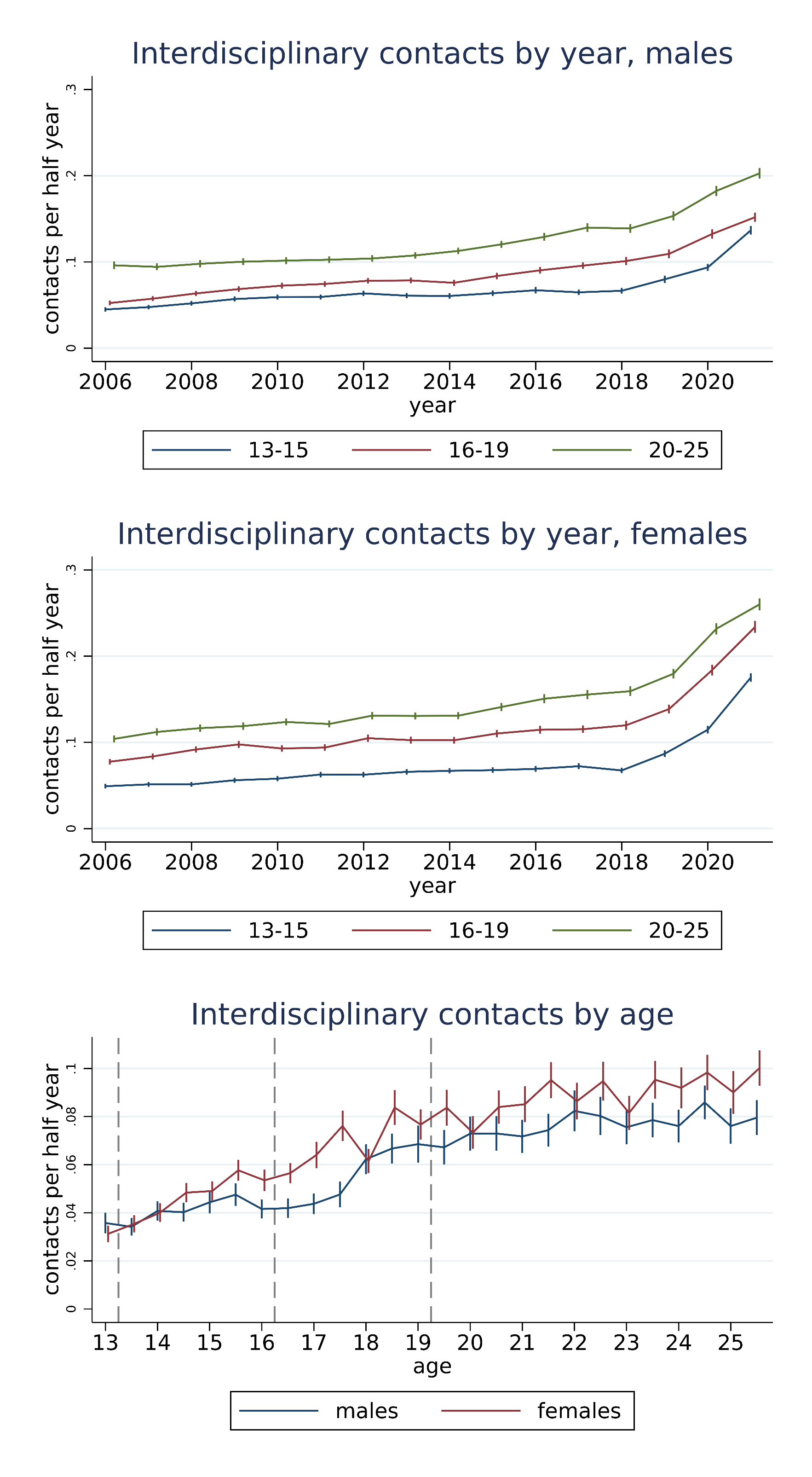


References

1. Statistics Norway. Statistical Classifications and Codelists [Internet] [cited 2023]. Available from: <https://www.ssb.no/en/klass/>
